# Medium-Chain Acyl-CoA Dehydrogenase Deficiency (MCADD) Newborn Screening in Italy: Five Years’ Experience from a Nationwide Program

**DOI:** 10.3390/ijns11040086

**Published:** 2025-09-26

**Authors:** Margherita Ruoppolo, Cristina Cereda, Teresa Giovanniello, Sabrina Malvagia, Sara Boenzi, Francesca Teofoli, on behalf of the SIMMESN Italian Newborn Screening Group, Alberto Burlina

**Affiliations:** 1 Department of Molecular Medicine and Medical Biotechnology, University of Naples, Federico II, 80131 Naples, Italy; 2 Ceinge Advanced Biotechnology Franco Salvatore Scarl, 80131 Naples, Italy; 3 Center of Functional Genomics and Rare Diseases, ‘V. Buzzi’ Children’s Hospital, 20154 Milan, Italy; cristina.cereda@asst-fbf-sacco.it; 4 Department of Experimental Medicine, Newborn Screening Center-Clinical Pathology Unit, University Hospital Policlinico Umberto I, Sapienza University, Rome, Italy, 00161 Rome, Italy; t.giovanniello@policlinicoumberto1.it; 5 Newborn Screening, Clinical Biochemistry and Clinical Pharmacy Laboratory, Meyer Children’s Hospital IRCCS, 50139 Florence, Italy; sabrina.malvagia@meyer.it; 6 Division of Metabolic Diseases and Hepatology, Bambino Gesù Children’s Hospital IRCCS, 00165 Rome, Italy; sara.boenzi@opbg.net; 7 Department of Surgery, Dentistry, Paediatrics and Gynaecology, University of Verona, 37129 Verona, Italy; francesca.teofoli@aovr.veneto.it; 8 Laboratory Medicine Service, Regional Center for Newborn Screening, Diagnosis and Treatment of Inherited Metabolic Diseases and Congenital Endocrine Diseases, Azienda Ospedaliera Universitaria Integrata of Verona, 37126 Verona, Italy; 9 Division of Inherited Metabolic Diseases, Department of Women’s and Children’s Health, University Hospital of Padua, 35128 Padua, Italy; alberto.burlina@unipd.it

**Keywords:** *ACADM* gene, *ACADM* variants, acylcarnitine, fatty acid oxidation disorder, LC-MS/MS, MCADD, newborn screening

## Abstract

Medium-chain acyl-CoA dehydrogenase deficiency (MCADD) is an autosomal recessive disorder of fatty acid oxidation that can have life-threatening consequences if not promptly treated. Early diagnosis by means of newborn screening (NBS) has the potential to reduce morbidity and mortality. This study investigates the incidence and molecular characteristics of MCADD in Italy over a five-year period within the framework of the expanded NBS program. Between January 2019 and December 2023, a total of 1,976,473 newborns were screened. Ninety unrelated neonates were diagnosed with MCADD, providing an estimated incidence of 1/21,960 live births (95% CI: 1:17,780–1:27,200), comparable to rates reported in other Mediterranean populations. Molecular analysis identified c.985A>G (p.Lys329Glu) as the most frequent pathogenic *ACADM* gene variant, observed in 56 patients (63%), including eighteen patients (20%) who were homozygous and thirty-eight (43%) who were compound heterozygotes for this variant. To our knowledge, this study represents the first comprehensive investigation to document the high prevalence of MCADD among the Italian population.

## 1. Introduction

Medium-chain acyl-CoA dehydrogenase (MCAD) deficiency (OMIM 201450) is the most common inherited disorder of fatty acid oxidation [1]. Encephalopathy and coma usually occur in infancy, associated with hypoketotic hypoglycemia. Early diagnosis markedly improves prognosis, particularly through the avoidance of prolonged fasting and providing the implementation of emergency protocols during catabolic states (e.g., intercurrent illnesses). The MCAD deficiency (MCADD) is inherited as an autosomal recessive trait, and it is caused by biallelic pathogenic variants in the *ACADM* gene, which maps on chromosome 1 (1p31) and comprises 12 exons, spanning about 44 kb.

Newborn screening for MCAD deficiency (MCADD) has been systematically implemented in many countries since the mid to late 1990s, as part of broader public health initiatives aimed at the early detection and management of inborn errors of metabolism. This approach seeks to prevent severe complications and improve long-term outcomes in affected infants [2,3,4]. The implementation of newborn screening (NBS) has also enabled more accurate estimates of MCADD incidence, now reported to range between 1 in 10,000 and 1 in 20,000 live births across various populations [5].

MCADD is mandatorily included in the Italian national neonatal screening program, following the enactment of laws between 2016 and 2017 (Law 167/2016; Ministerial Decree of 13 October 2016; Decree of the President of the Council of Ministers of 12 January 2017) [6]. However, as early as 2008, some Italian regions had independently introduced MCADD screening within their own local programs.

Prior to the implementation of expanded NBS, MCADD was considered an extremely rare disorder in Southern European countries [7]. In Italy, the first case was reported in 1995 [8], and only three symptomatic cases were identified over a 13-year period (1985–1997), as reported in the retrospective study by Dionisi-Vici et al. [9]. This highlights the limited diagnostic capacity in the absence of systematic screening protocols.

Unfortunately, no national disease registry for MCADD was established in Italy prior to 2018, resulting in limited reliable data on the number of symptomatic patients, total births, or clinical outcomes during the pre-screening period. However, reports from the Italian Society for the Study of Hereditary Metabolic Diseases and Newborn Screening (SIMMESN) indicate that between 2008 and 2017, 88 cases of MCADD were identified among approximately 1,724,600 newborns screened across regions that had independently implemented screening programs, suggesting an incidence consistent with our findings in the 2019–2023 cohort.

To evaluate the effectiveness of the Italian NBS program for MCADD, we conducted a nationwide retrospective observational study, analyzing both screening and confirmatory data collected from 2019 to 2023. An incidence of 1:21,960 live births (95%CI: 1/17,780–1:27,200) was estimated, comparable to rates reported in other Mediterranean populations.

Molecular analysis identified c.985A>G (p.Lys329Glu) as the most frequent pathogenic variant. The c.985A>G *ACADM* gene variant, located in exon 11, leads to a lysine-to-glutamic acid substitution at position 329 of the medium-chain acyl-CoA dehydrogenase enzyme. Functional studies have demonstrated that the p.Lys329Glu substitution significantly reduces enzyme stability and activity, impairing mitochondrial fatty acid oxidation. The high prevalence of this gene variant in diverse populations may be explained by a common ancestral origin and a potential founder effect. Notably, individuals homozygous for c.985A>G tend to exhibit more severe biochemical profiles, although clinical heterogeneity can be present.

## 2. Materials and Methods

### 2.1. Ethics Approval

This retrospective cohort study was approved by the Ethics Committee Campania 3 of the University of Naples “Federico II” (Protocol No. 161/2024 approved on 24 June 2024).

### 2.2. NBS Protocol

Dried blood spots (DBS) were collected between 48 and 72 h after birth and sent to one of the 16 national screening laboratories for analysis. The organizational structure of the Italian NBS program has been described in detail elsewhere [6]. National laboratories are responsible for quality monitoring to ensure the accuracy, reliability, and consistency of the screening results. This includes regular performance evaluation and participation in external quality assurance programs using DBS quality controls, provided by SIMMESN, European Research Network for Inherited Disorders of Metabolism (ERNDIM) and the Centers for Disease Control and Prevention (CDC, Atlanta, GA, USA).

The most commonly used method was underivatized sample preparation, due to its lower time requirements. At the time of the study, two laboratories employed derivatization to butyl esters for sample preparation. Most laboratories used certified commercial kits, while two laboratories employed internally validated in-house methods with isotope-labeled internal standards provided by Cambridge Isotope Laboratories (CIL).

The most commonly used mass spectrometer was the Waters Xevo TQD (Waters Corporation, Milford, MA, USA). Other laboratories used Perkin Elmer Qsight (Waltham, MA, USA) and AB Sciex Systems (Framingham, MA, USA).

C6, C8, C10 and C10:1 acylcarnitines were used as primary biomarkers, while the C8/C10 and C8/C2 ratios served as secondary biomarkers. Screening protocols varied among NBS laboratories, regarding of the cut-off values applied. One NBS laboratory applied the CLIR system (Collaborative Laboratory Integrated Reports, Mayo Clinic, Rochester, MN, USA), using post-analytical tools to identify abnormal acylcarnitine profiles, independently of predefined cut-off values.

In case of abnormal results, the screening test was repeated on the residual dried blood spot to confirm the initial finding (retesting o repeat test). Newborns with abnormal values were referred to the regional referent clinic center for metabolic disorders for further clinical evaluations and confirmatory diagnostic testing.

Diagnostic confirmation relied on a combination of several tests, including plasma acylcarnitine profile, molecular genetic analysis, and the assessment of MCAD enzyme activity.

Measurement of MCAD residual activity was performed in lymphocytes according to the previously described protocol [10]. Residual activity is expressed as a percentage of wild-type (WT) levels.

### 2.3. Genetic Analysis

Although MCADD is caused by mutations in a single gene, the *ACADM* gene, the use of either Sanger sequencing or NGS depended on local protocols and the technological infrastructure of each screening laboratory. Sanger sequencing, a widely adopted method, was used to detect specific variants in the *ACADM* gene. Alternatively, Next-Generation Sequencing (NGS), enabled a more comprehensive, high-throughput analysis, allowing also the identification of Copy Number Variations (CNVs). In addition, few laboratories used Multiplex ligation-dependent probe amplification (MLPA), as a complementary method to detect CNVs in the *ACADM* gene.

Variant classification was performed according to guidelines of the American College of Medical Genetics and Genomics (ACMG) guidelines [11]. The archives of ClinVar (https://www.ncbi.nlm.nih.gov/clinvar/, accessed 10 July 2025), VarSome (https://varsome.com/, accessed 10 July 2025) [12], Franklin (https://franklin.genoox.com, accessed 10 July 2025) (DOI: 10.1093/bioinformatics/bty897) and HGMD professional 2025.1 (Human Gene Mutation Database, Professional 2025.1) were also consulted. To date, over 200 pathogenic and likely pathogenic variants have been reported, along with nearly 70 variants of uncertain significance (VUS).

Pathogenic and likely pathogenic variants were used to define genetically confirmed cases, in accordance with the ACMG guidelines (DOI: 10.1038/gim.2015.30) using the RefSeq transcript number NM_000016.6. [11]. In addition, cases harboring a novel variant or a variant of uncertain significance (VUS) identified in trans with a pathogenic or likely pathogenic variant were also considered genetically positive when supported by concordant biochemical findings.

### 2.4. Statistical Analysis

Statistical analyses were performed using GraphPad Prism 9 software (GraphPad Software, Boston, MA, USA). Normality of data distribution was assessed using D’Agostino tests. For non-normally distributed variables, descriptive statistics included minimum and maximum values, median, first quartile (Q1), and third quartile (Q3). Data are presented as individual data points with medians and interquartile ranges.

The reliability of the mean estimate was assessed by computing the 95% confidence interval (CI).

For non-normally distributed variables, group comparison was conducted using the Kruskal–Wallis test, followed by Dunn’s post hoc test for multiple comparisons. Statistical significance was defined as follows: *p*-value < 0.05 (*), *p*-value < 0.01 (**), *p*-value < 0.001 (***), *p*-value < 0.0001 (****) and ns (not significant).

To assess associations between DBS acylcarnitine levels and residual enzymatic activity, Spearman’s rank correlation was applied with a two-tailed test and 95% CI. A *p*-value of <0.05 was considered statistically significant.

## 3. Results

Between 2019 and 2023, a total of 1,976,473 newborns were screened for MCADD in Italy across 16 designated regional newborn screening laboratories. DBS samples were collected at a median age of 2.4 days, in agreement with Italian law, and screening analyses were performed at a mean age of 4.1 days. Newborns with abnormal screening results were referred to the regional referent clinical centers for further confirmatory evaluation within the first week of life.

### 3.1. MCADD Confirmed Newborns

Among the 1,976,473 newborns screened, 99 were identified as biochemically positive for MCADD. Of these, 90 cases were genetically confirmed. The remaining nine cases included: three had only one *ACADM* gene variant, one with no variations detected, three with molecular results not reported in the data collection, one lost to follow-up and one with two variants of uncertain significance (VUS).

In the three cases with only one variation identified, further investigations are ongoing, based on markedly elevated acylcarnitine levels observed both at screening and during follow-up.

Two genetically confirmed cases were excluded from the study due to lack of parental consent for inclusion of their data in the national study; however, they were included in the incidence calculation. Accordingly, the following analyses refer to a final cohort of eighty-eight genetically confirmed MCADD cases.

Based on 1,976,473 live births and 90 confirmed cases (including the two excluded from detailed analysis due to lack of parental consent), the estimated incidence of MCAD deficiency in Italy was 1:21,960 live births (95% CI: 1:17,780–1:27,200). This incidence is comparable to rates reported in other Mediterranean populations and significantly higher than previously estimated for Southern European countries.

### 3.2. Demographic and Clinical Characteristics

The majority of the eighty-eight MCAD-positive newborns were of European geographic origin. Minority backgrounds included four of Asian origin, one from South America, and three from North Africa. The median birth weight of MCADD newborns was 3.3 kg. Two newborns were preterm and classified as very low birth weight infants. Among the cohort, forty-two were males and forty-six were females.

Most infants were delivered vaginally, while twenty-two (25%) were born via cesarean section. Feeding practices at discharge included exclusive breastfeeding in sixty cases, mixed feeding (breast milk and formula) in twenty-three cases and exclusively formula-fed in five cases.

Seven infants experienced hypoglycemia at birth (glucose levels ranging from 1.3 to 2.5 mmol/L), occurring within the first 24–48 h of life. The hypoglycemic episodes were generally mild and responded well to glucose supplementation. Three infants required intravenous glucose administration, while the others were managed with frequent feeding protocols. In some cases, hypoglycemia developed within the first day of life and was associated with severe clinical presentations including hypotonia, abnormal eye movements, cardiac arrest, or combined hypothermia and supraventricular tachycardia.

At the time of the recall visit, all but nine newborns were asymptomatic. A positive family history for MCADD (siblings or cousins) was reported in eighteen cases. These family members had been identified as MCADD cases through previous newborn screening programs, confirming the familial nature of the disorder. Table 1 shows statistical values for biochemical-clinical parameters recorded at the first metabolic visit in the regional referral clinic center after notification of the positive screening.

### 3.3. Acylcarnitine Profile in NBS Analysis

**Figure 1:**
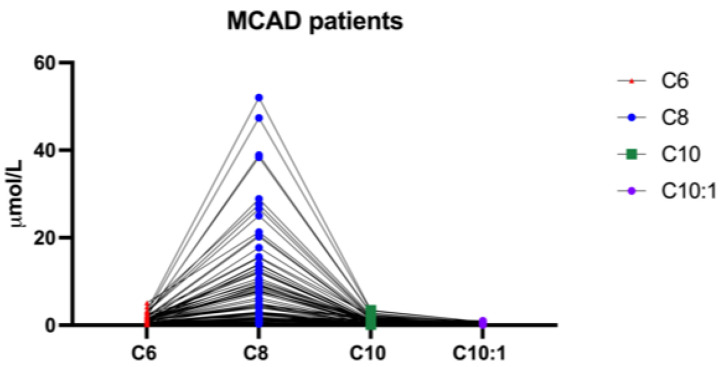
Distribution of dried blood spot C6, C8, C10, and C10:1 in the confirmed positive newborns, showing the typical MCADD pattern with predominant C8 elevation and milder increases in C6, C10, and C10:1. The exact concentration values for C6, C10, and C10:1 are reported in Table 2, Table 3, Table 4 and Table 5.

Table 2, Table 3, Table 4 and Table 5 show dried blood spot values for the most relevant acylcarnitine biomarkers in the cohort of eighty-eight confirmed MCADD cases. Reference ranges of blood acylcarnitines are calculated based on the mean ± SD from data collected across all screening centers and are expressed in µmol/L. Free carnitine (n.v. 6.6 ± 1.3–54.2 ± 10.2); C2 (n.v. 6.4 ± 2.6–54.3 ± 13.0); C6 (n.v. ≤ 0.2 ± 0.1); C8 (n.v. ≤ 0.3 ± 0.1); C10 (n.v. ≤ 0.3 ± 0.1); C10:1 (n.v. ≤ 0.2 ± 0.1);

Figure 1 illustrates the distribution patterns of dried blood spot biomarkers C6, C8, C10, and C10:1 in these newborns. In all cases, the four biomarkers exhibited a characteristic “mountain-shaped” profile, with C8 levels consistently exceeding those of the other analytes. Plasma acylcarnitine analysis was performed on all referred cases, except for 15 newborns, in whom only an additional DBS specimen was available for confirmatory testing.

### 3.4. Enzyme Activity

MCAD enzyme activity data were available for 58% of newborns with positive NBS results (51/88). Among these (Figure S1), 31 newborns exhibited enzyme activity levels below 10% indicating a severe form of the disease in 60.7% of cases with available data, while the remaining 39.2% showed higher activity levels, consistent with a milder form of the disease.

**Figure 2:**
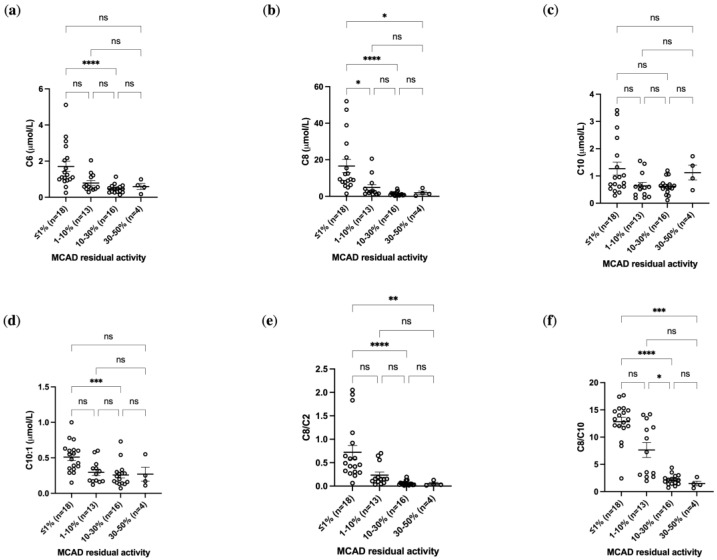
Distribution of acylcarnitine levels on DBS samples from subjects stratified by MCAD residual activity expressed as percentage of wt. Plots summarizing. (**a**) C6 concentrations, (**b**) C8 concentrations, (**c**) C10 concentrations, (**d**) C10:1 concentrations, (**e**) C8/C2 ratios and (**f**) C8/C10 ratios in different MCAD residual activity groups of subjects with enzymatic activity < 1% (*n* = 18), between 1 and 10% (n = 13), between 10 and 30% (n = 16), between 30 and 50% (N = 4). Significant differences were established by performing Kruskal–Wallis and Dunn’s multiple comparison tests (* *p* < 0.05, ** *p* < 0.01, *** *p* < 0.001 **** *p* < 0.0001, ns = not significant). The Spearman ρ correlation values between C6 and MCAD residual activity (ρ = −0.3286, *p* = 0.0186), C8 and MCAD residual activity (ρ = −0.4580, *p* = 0.0007), C10 and MCAD residual activity (ρ = −0.1695, *p* = 0.2345), and C10:1 and MCAD residual activity (ρ = −0.3846, *p* = 0.0053) were evaluated. The strongest correlations were observed for C8 levels (ρ = −0.4580, *p* = 0.0007), indicating that C8 concentrations in DBS samples are reliable predictors of residual MCAD enzyme activity.

A correlation analysis was carried out between DBS acylcarnitine levels and residual enzyme activity. As shown in Figure 2, higher residual enzyme activity was associated with lower concentrations of C6 and C8 in DBS samples, consistently with previously published data [10]. Newborns with residual enzyme activity between 0 and 10% showed the highest levels of C6 (up to 5.11 µmol/L) and C8 (up to 52.03 µmol/L). Furthermore, significant differences in concentrations of C6 (*p*-value < 0.0001), C8 (*p*-value < 0.0001) and C10:1 (*p*-value < 0.001) were observed between groups with enzymatic activity <1% and those with activity between 10 and 30%. In contrast, no significant differences were found for C10 levels (Figure 2) and free carnitine level, C0 (Figure S2). It can be noted that the statistical significance of the comparison between the group with enzimatic activity < 1% and that one with enzimatic activity between 30 and 50% is greater if the ratios of C8 are considered instead of C8 alone, in agreement with previous findings [10].

### 3.5. Gene Mutation Analysis

Gene variation analysis revealed substantial genotypic variability observed within the cohort (Table 2, Table 3, Table 4 and Table 5). Among the eighty-eight confirmed cases, 27 individuals (30%) were homozygous and 61 were compound heterozygotes (69%). A total of 44 different *ACADM* gene variants were identified, including 27 previously described and 16 novel variants.

The most prevalent variant was c.985A>G (p.Lys329Glu) found in 56 out of 88 patients. Of these, 18 (20%) were homozygous for the variant while 38 (43%) were compound heterozygotes.

**Figure 3:**
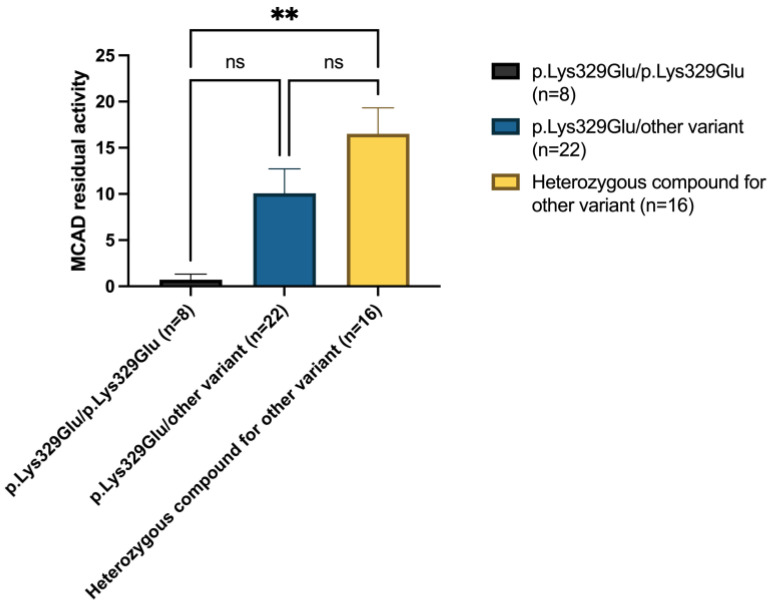
The distribution of the range of residual activity and molecular genetic analysis. Plots represent MCAD residual activity in three different genetic groups of patients. Significant differences were established by performing Kruskal–Wallis and Dunn’s multiple comparison tests (* *p* < 0.05, ** *p* < 0.01, *** *p* < 0.001 **** *p* < 0.0001, ns = not significant).

Figure 3 illustrates the relationship between residual enzymatic activity and genotype. Patients homozygous for the c.985A>G variant showed significantly lower residual MCAD activity compared to patients compound heterozygous for two different *ACADM* variants other than c.985A>G (*p*-value < 0.001). Among patients carrying c.985A>G in trans with another variant, enzymatic activity levels were intermediate, suggesting a correlation between genotype and residual function.

**Figure 4:**
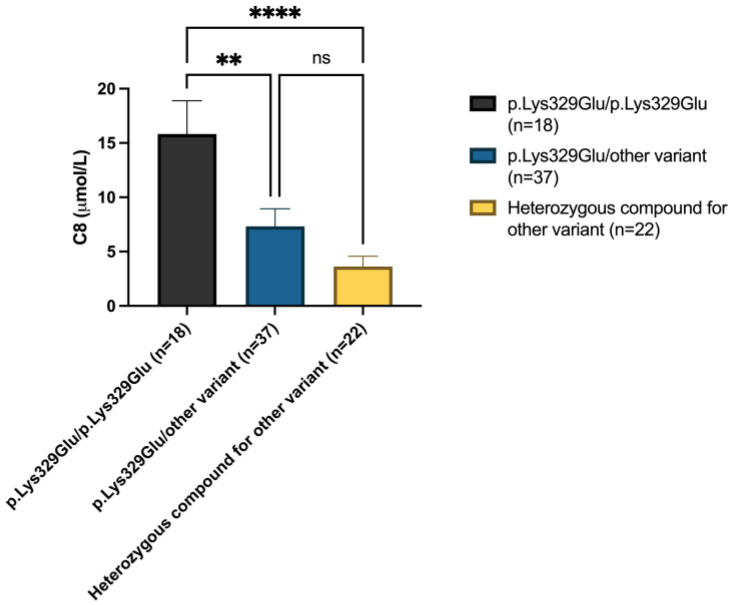
C8-carnitine was significantly higher in homozygotes for the p.Lys329Glu pathogenic variant than in heterozygotes when measured in newborn screening blood spots. Significant differences were established by performing Kruskal–Wallis and Dunn’s multiple comparison tests (* *p* < 0.05, ** *p* < 0.01, *** *p* < 0.001 **** *p* < 0.0001, ns = not significant).

We investigated the relationship between genotype and octanoyl-carnitine (C8) levels measured in neonatal DBS. Surprisingly, in individuals homozygous for the c.985A>G (p.Lys329Glu) gene variant, C8 concentration showed a wide range (Figure 4), significantly higher than that measured in the two groups of heterozygous compounds.

Detailed neonatal screening data for this subgroup are provided in Table 4, including C8, C8/C2, and C8/C10 ratios, as well as the presence, timing, and severity of hypoglycemia or other symptoms within the first 48 h of life. This table highlights that, while most homozygous newborns remained asymptomatic in the early neonatal period, some developed hypoglycemia within the first day of life, occasionally accompanied by severe manifestations such as hypotonia, abnormal eye movements, cardiac arrest, or combined hypothermia and supraventricular tachycardia. These findings further underline the wide biochemical and clinical variability observed even among patients sharing the same genotype.

**Figure 5:**
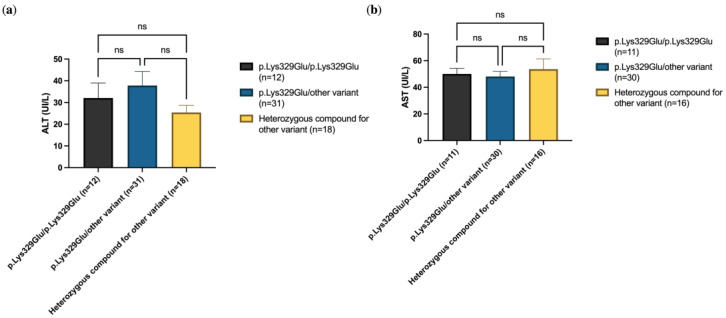
ALT (**a**) and AST (**b**) were not significantly different in p.Lys329Glu homozygotes as compared to heterozygotes. Significant differences were established by performing Kruskal–Wallis and Dunn’s multiple comparison tests (* *p* < 0.05, ** *p* < 0.01, *** *p* < 0.001 **** *p* < 0.0001, ns = not significant).

Previous studies have reported that individuals homozygous for the c.985A>G (p.Lys329Glu) gene variant display significantly elevated transaminase levels (ALT and AST) compared to compound heterozygotes [13]. However, in our cohort, we did not observe any statistical significance (Figure 5).

## 4. Discussion

NBS has markedly improved clinical outcomes for infants diagnosed with MCAD deficiency by enabling timely dietary intervention, appropriate medical management, and targeted caregiver education. Importantly, mortality associated with MCADD has been virtually eliminated when the condition is identified through NBS.

In Italy, MCADD is included in the NBS program for inborn errors of metabolism, which was officially established by law between 2016 and 2017. Here we present data collected from 2019 to 2023. Based on a population of 1,976,473 live births and 88 confirmed cases, the estimated incidence of MCAD deficiency in Italy was 1 in 22,460 consistent with previous national data [6,14]. Including two additional confirmed cases of MCAD, excluded from this study due to lack of parental consent, the estimated incidence increases to 1 in 21,960 live births (95%CI: 1/17,780–1:27,200).

Similarly, in other Mediterranean countries, the implementation of long-term NBS has demonstrated clear benefits in early detection and prevention of severe clinical outcomes. Reported incidences include 1 in 16,951 in Galicia [15], 1 in 13,787 in the Madrid region [16], and 1 in 17,066 in Turkey [17]. Higher prevalences have been observed in Portugal (1 in 6603) [18] and North Macedonia (1 in 4813) [19].

To date, no false-negative cases have been reported in Italy during the study period. Furthermore, no instances of fatal metabolic decompensation were observed prior to receipt of NBS results or before NBS sampling. However, between 2016 and 2018, two newborns developed severe symptoms with fatal outcomes before NBS results became available (Burlina, A. personal communication). The findings from recent research on sudden neonatal death in individuals with MCADD [20] highlight the limitations of NBS in identifying the most severe cases with onset that anticipates the timing of newborn screening.

The data presented in this study clearly demonstrate that functional testing to quantify residual MCAD activity is helpful for phenotype prediction. Our results show a strong correlation between initial C6 and C8 acylcarnitine levels detected by NBS and residual enzyme activity. Specifically, the highest concentration of C6 and C8 was observed in patients with the lowest enzyme function. This finding aligns with previous studies [10,21] that emphasized the role of enzyme activity in differentiating between severe and milder forms of MCADD, particularly in cases involving novel genetic variants.

Early identification of severe phenotypes through NBS has markedly improved the clinical course of the disease by enabling the prevention of hypoglycemic crises that could otherwise jeopardize neonatal health. Importantly, our study also highlights the presence of milder phenotypes, characterized by relatively higher residual enzyme activity, which account for a substantial proportion (approaching 40%) of the MCADD population. By ‘milder phenotype,’ we refer to individuals with residual MCAD enzyme activity greater than 10%, who may exhibit less severe biochemical abnormalities and potentially a lower risk of metabolic decompensation. However, it is important to note that all identified cases are managed with preventive measures, including emergency protocols, which complicates direct comparisons of clinical severity in the context of newborn screening.

This phenotypic variability has a direct impact on incidence estimates and raises ongoing questions about the clinical significance of MCADD cases with moderate or high residual enzyme activity. Whether such individuals are truly at risk of metabolic decompensation remains a subject of debate [10].

Molecular confirmation of positive NBS results has significantly contributed to a deeper understanding of the genetic heterogeneity underlying MCADD. In our cohort of 88 genetically confirmed MCADD patients, the most common variant was c.985A>G (p.Lys329Glu), identified in 56 individuals (63%), either in homozygous or compound heterozygous form. Based on genotype distribution, the allele frequency of this variant was estimated at 42.0% (74 of 176 alleles) in affected individuals. Based on NBS incidence and allele distribution, the population allele frequency of c.985A>G in Italy can be estimated at approximately 0.3% (95% CI: 0.23–0.34%). According to gnomAD (v4), the global allele frequency of the c.985A>G variant is approximately 0.6%, while in the European (non-Finnish) population, it is around 0.7%.

Comparable findings have been reported in other Mediterranean regions, including Galicia, Portugal, and North Macedonia, where p.Lys329Glu also emerged as the most frequent variant among diagnosed patients [15,18,19]. These data support the idea that population-specific founder effects or underestimated carrier frequencies may contribute to regional variability in MCADD presentation. These findings support the hypothesis of potential founder effects in Southern European populations [22]. From a public health perspective, this finding may inform carrier screening strategies, genetic counseling, and the interpretation of VUS in Southern European populations.

We investigated the relationship between genotype and C8 acylcarnitine levels at the time of NBS. Newborns homozygous for the c.985A>G (p.Lys329Glu) variant showed a wide range of C8 concentrations, which were significantly different from those observed in compound heterozygotes. The investigation of the relationship between genotype and residual MCAD activity showed that patients homozygous for the c.985A>G variant showed significantly lower residual MCAD activity compared to compound heterozygotes for two different *ACADM* variants other than c.985A>G.

While advances in newborn screening and genetic diagnostics have significantly improved the early detection and management of MCADD, important challenges remain in clinical care and the development of effective therapies. As highlighted in recent reviews, including comprehensive analyses of mitochondrial fatty acid oxidation disorders, substantial knowledge gaps persist, particularly regarding disease pathophysiology and long-term treatment strategies [1]. A recent nationwide study from Czechia highlighted the significant impact of ENBS on diagnostic rates and clinical outcomes in patients with MCADD [23]. Interestingly, in our Italian cohort, the predominance of the severe c.985A>G variant suggests that the increased incidence following expanded NBS implementation reflects improved ascertainment of both severe and milder genotypes, as reported in other European countries with NBS programs [23,24,25]. This finding contrasts with some studies that primarily reported increased detection of milder variants post-NBS, highlighting potential population-specific differences in MCADD genetic architecture. Given the lack of robust pre-NBS national data, we prefer to cautiously state that the observed incidence in Italy likely reflects a combination of improved ascertainment of both mild and severe genotypes, together with a persistently high background frequency of high-impact variants, as reported in other European cohorts.

A major limitation of the present study is the lack of long-term follow-up data to assess clinical outcomes over time. To address this gap, we plan a dedicated longitudinal study aimed at evaluating long-term outcomes, treatment efficacy, and potential complications in individuals diagnosed with MCADD through NBS. Furthermore, the absence of structured national disease registries in Italy during the pre-screening era hampers the reconstruction of historical incidence and the clinical burden of symptomatic MCADD. This underscores the urgent need to establish systematic, long-term registries for inborn errors of metabolism, which would complement NBS programs by providing longitudinal data on clinical outcomes, disease natural history, and the real-world effectiveness of management protocols.

The systematic collection of biochemical and genetic data from large patient cohorts, shared through international networks, is essential to advance the principles of predictive, preventive, personalized, and participatory (P4) medicine. Such data are critical for supporting the development of targeted therapeutic strategies. In this context, the involvement of European Reference Networks such as MetabERN (https://metab.ern-net.eu), and the integration of national datasets into pan-European platforms like the Unified European Registry for Inherited Metabolic Diseases (U-IMD) (https://u-imd.org), represent key steps toward harmonized long-term follow-up and improved patient care across Europe.

## 5. Conclusions

This study demonstrates that NBS using LC-MS/MS is highly effective for early MCADD diagnosis in the Italian population, with an estimated incidence of 1:21,960 live births (significantly higher than previously recognized). The predominance of the severe c.985A>G variant in our cohort underscores the clinical importance of NBS implementation, as early identification enables timely preventive interventions that have virtually eliminated MCADD-related mortality. These findings provide crucial data for healthcare planning and genetic counseling in Mediterranean populations and support the need for robust national registries to monitor long-term outcomes. The expanded implementation of NBS has also enabled the identification of a wide array of *ACADM* gene variants, thereby improving our understanding of the genetic basis and phenotypic variability of the disorder. In our cohort, the c.985A>G (p.Lys329Glu) variant emerged as the most common mutated allele, detected in both homozygous and compound heterozygous states.

## Figures and Tables

**Table 1:** Summary of Laboratory Data in MCADD Patients at time of diagnosis.

Parameter	Patients with Data	Reference Range	Min	Max	Median	1Q	3Q
AST (Aspartate Transaminase)	65	<70 U/L	18.0	137	45.0	35.5	59.0
ALT (Alanine Transaminase)	71	<55 U/L	9.0	179	24.0	18.0	36.0
Uric Acid	35	120–480 µmol/L	82.7	7440	190	131	880
Ammoniemia	33	<80–100 µmol/L	11.7	85.0	33.9	21.4	49.7
CPK (Creatine Phosphokinase)	61	<300 U/L	46.0	1480	142	105	260

All values measured in plasma samples collected at first metabolic evaluation following positive newborn screening. Reference ranges are age-appropriate for neonates.

**Table 2:** Biochemical newborn screening data in infants homozygous for the ACADM p.Lys329Glu variant.

Patient	Origin	Country	Sex	Free Carnitine (µmol/L)	C2 (µmol/L)	C6 (µmol/L)	C8 (µmol/L)	C10 (µmol/L)	C10:1 (µmol/L)	Symptoms Before 48 h of Life	Blood Glucose at Recall (n.v. 2.8–6.1 mmol/L)
P1	European	Italy	F	27.11	19.60	0.25	8.93	0.74	0.43	NO	NA
P2	European	Italy	M	27.41	21.69	0.66	13.07	1.20	0.67	NO	NA
P3	European	Romania	M	15.93	11.68	0.47	4.53	0.40	0.32	Hypotonia; intermittent moaning; abnormal eye movements	NA
P4	European	Italy	M	16.07	23.13	1.44	9.12	1.14	0.50	NO	NA
P5	European	Italy	M	17.96	23.98	2.04	15.41	1.32	0.55	NO	4.9
P6	European	Italy	F	16.89	19.85	0.89	5.74	0.39	0.34	NO	5.1
P7	Asian	Pakistan	F	14.55	23.08	2.82	47.35	3.27	0.76	hypoglycemia, hypotonia, hypothermia, and supraventricular tachycardia,	2.0
P8	European	Italy	M	23.60	31.80	0.39	2.70	0.20	0.22	NO	2.3
P9	European	Italy	M	13.80	25.40	3.10	28.90	2.40	0.60	NO	3.8
P10	European	Albania	M	14.62	21.90	2.34	38.38	2.67	0.68	NO	3.9
P11	European	Italy	M	13.38	20.12	1.11	1.89	0.61	0.17	Mild acidosis and hypoglycemia on the first day of life, resolved with the administration of breast milk.	4.5
P12	European	Romania	F	20.21	23.15	5.11	21.29	1.75	1.00	NO	5.5
P13	European	Italy	M	16.03	23.17	1.42	10.16	1.30	0.31	NO	2.8
P14	European	Italy	M	20.18	12.59	1.36	8.24	1.27	0.33	Severe neonatal hypoglycemia, cardiac arrest	21.4 *
P15	European	Kosovo	M	27.05	23.22	0.96	4.15	0.38	0.21	Neonatal hypoglycemia	NA
P16	European	Romania	M	20.70	27.58	3.04	25.00	1.67	0.53	NO	4.2
P17	European	Romania	F	18.70	17.00	4.22	27.57	2.01	0.65	NO	3.8
P18	European	Italy	F	14.0	21.0	1.6	12.48	0.94	0.63	NO	4.4

* The highest blood glucose value was from a newborn monitored after an emergency glucose infusion. Not available: enzyme activity measurement not performed. Reference ranges of blood acylcarnitines are expressed in µmol/L. Free carnitine (n.v. 6.6 ± 1.3–54.2 ± 10.2); C2 (n.v. 6.4 ± 2.6–54.3 ± 13.0); C6 (n.v. ≤ 0.2 ± 0.1); C8 (n.v. ≤ 0.3 ± 0.1); C10 (n.v. ≤ 0.3 ± 0.1); C10:1 (n.v. ≤ 0.2 ± 0.1).

**Table 3:** Biochemical newborn screening data in infants homozygous for the ACADM gene (NM_000016.6/NP_000007.1).

Patient	Origin	Country	Sex	Free Carnitine (µmol/L)	C2 (µmol/L)	C6 (µmol/L)	C8 (µmol/L)	C10 (µmol/L)	C10:1 (µmol/L)	Symptoms Within 48 h of Life	Allele 1	Allele 2	ACMG	Enzyme Activity %	Blood Glucose at Recall (n.v. 2.8–6.1 mmol/L)
P19	European	Albania	M	22.5	25.6	0.93	7.78	0.44	0.35	NO	c.242-243insT p.Trp82Leufs*23	c.242-243insT p.Trp82Leufs*23	P/P	0	NA
P20	European	Albania	F	20.95	14.48	0.42	2.69	0.2	0.27	NO	c.242-243insT p.Trp82Leufs*23	c.242-243insT p.Trp82Leufs*23	P/P	2	NA
P21	European	Albania	F	11.1	12.2	1.4	13.8	1.2	0.74	NO	c.242-243insT p.Trp82Leufs*23	c.242-243insT p.Trp82Leufs*23	P/P	NA	NA
P22	European	Albania	M	10.11	12.32	1.21	17.73	1.75	0.47	NO	c.242-243insT p.Trp82Leufs*23	c.242-243insT p.Trp82Leufs*23	P/P	NA	3.3
P23	European	Italy	M	18.39	21.22	2.17	38.89	2.78	0.61	Hypoglycemia	c.257dup p.Leu86PhefsTer19	c.257dup p.Leu86PhefsTer19	P/P	0	NA
P24	Asian	Turkiye	M	36.34	24.86	1.04	3.92	0.56	0.42	NO	c.799G>A p.Gly267Arg	c.799G>A p.Gly267Arg	P/P	NA	5.4
P25	Asian	Pakistan	M	34.72	45.64	1.31	7.55	1.31	0.42	NO	c.799G>A p.Gly267Arg	c.799G>A p.Gly267Arg	P/P	NA	4.3
P26	European	Italy	M	22.13	40.66	1.84	7.63	1.41	0.49	NO	c.388-14A>G	c.388-14A>G	VUS/VUS ***	NA	4.4
P27	European	Romania	M	20.33	20.04	0.23	0.72	0.85	0.27	NO	c.199T>C Tyr67His	c.199T>C Tyr67His	P/P	NA	NA

Novel variants are indicated by underlining, and their ACMG/AMP classification is provided in the Table. *** Potential Splicing Impact. Not available: enzyme activity measurement not performed. Reference ranges of blood acylcarnitines are expressed in µmol/L. Free carnitine (n.v. 6.6 ± 1.3–54.2 ± 10.2); C2 (n.v. 6.4 ± 2.6–54.3 ± 13.0); C6 (n.v. ≤ 0.2 ± 0.1); C8 (n.v. ≤ 0.3 ± 0.1); C10 (n.v. ≤ 0.3 ± 0.1); C10:1 (n.v. ≤ 0.2 ± 0.1).

**Table 4:** Biochemical newborn screening data in infants compound heterozygous for the ACADM c.985A>G variant and a second ACADM variant (NM_000016.6/NP_000007.1).

Patient	Origin	Country	Sex	Free Carnitine (µmol/L)	C2 (µmol/L)	C6 (µmol/L)	C8 (µmol/L)	C10 (µmol/L)	C10:1 (µmol/L)	Symptoms Within 48 h of Life	Allele 1	Allele 2	ACMG	Enzyme Activity %	Blood Glucose at Recall (n.v. 2.8–6.1 mmol/L)
P28	European	Italy	F	26.22	21.08	0.55	2.87	0.49	0.35	NO	c.985A>G p.Lys329Glu	c.184C>T p.Pro62Ser	P/VUS	NA	NA
P29	European	Italy	F	23.5	9.21	0.49	1.38	0.19	0.16	NO	c.985A>G p.Lys329Glu	c.253G>C p.Gly85Arg	P/P	9.8	4.9
P30	European	Italy	F	20	15.9	0.27	0.6	0.18	0.09	NO	c.985A>G p.Lys329Glu	c.1247T>C p.Ile416Thr	P/VUS	NA	3.6
P31	European	Italy	F	16.9 ^#^	7.65 ^#^	0.28 ^#^	0.25 ^#^	0.06 ^#^	0.07 ^#^	NO	c.985A>G p.Lys329Glu	c.1247T>C p.Ile416Thr	P/VUS	NA	3.6
P32	European	Italy	M	29.2	29.31	2.04	20.57	1.45	0.6	NO	c.985A>G p.Lys329Glu	c.134A>G p.Gln45Arg	P/P	3.4	3.9
P33	European	Italy	F	29.8	20.2	0.31	1.19	0.56	0.28	NO	c.985A>G p.Lys329Glu	c.134A>G p.Gln45Arg	P/P	17.1	3.3
P34	European	Italy	M	48.7	48.73	0.58	2.3	0.65	0.31	Hypoglycemia	c.985A>G p.Lys329Glu	c.928G>A p.Gly310Arg	P/LP	4.6	1.3
P35	European	Italy	F	14.6	14.3	0.56	1.53	0.57	0.18	NO	c.985A>G p.Lys329Glu	c.734C>T p.Ser245Leu	P/P	6.1	1.3
P36	European	Italy	M	22.6	23.58	0.57	1.48	0.61	0.15	NO	c.985A>G p.Lys329Glu	c.734C>T p.Ser245Leu	P/P	0.0	2.0
P37	European	Italy	M	12.6	16.2	0.46	1.12	0.33	0.12	NO	c.985A>G p.Lys329Glu	c.734C>T p.Ser245Leu	P/P	4.40	NA
P38	European	Italy	M	16.53	13.45	0.47	1.49	0.34	0.21	NO	c.985A>G p.Lys329Glu	c.734C>T p.Ser245Leu	P/P	NA	NA
P39	European	Italy	F	41.3	35.2	1.1	7.9	0.53	0.48	NO	c.985A>G p.Lys329Glu	c.1102_1105delTTAG Ala369fs	P/P	0.0	5.1
P40	European	Italy	F	13.5	20.3	0.17	0.31	0.48	0.11	NO	c.985A>G p.Lys329Glu	c.1091T>C p.Ile364Thr	P/VUS	NA	4.8
P41	European	Italy	M	31.1	45.1	0.66	1.7	1.38	0.25	NO	c.985A>G p.Lys329Glu	c.797A>G p.Asp266Gly	P/LP	31.7	4.2
P42	European	Italy	M	29.23	29.32	0.26	0.77	0.54	0.13	NO	c.985A>G p.Lys329Glu	c.797A>G p.Asp266Gly	P/LP	28	4.3
P43	European	Italy	F	22.4	40.8	1.67	11.96	0.85	0.67	NO	c.985A>G p.Lys329Glu	c.977T>C p.Met326Thr	P/P	NA	4.8
P44	European	Italy	F	10.1	10.8	1.18	14	0.94	0.36	NO	c.985A>G p.Lys329Glu	c.27C>G p.Cys9Trp	P/VUS	NA	4.3
P45	European	Italy	F	8.77	13.71	2.37	10.74	0.99	0.33	NO	c.985A>G p.Lys329Glu	c.242-243insT p.Trp82Leufs*23	P/P	NA	5.5
P46	European	Italy	M	18.94	8.36	0.99	4.88	0.28	0.38	NO	c.985A>G p.Lys329Glu	c.242-243insT p.Trp82Leufs*23	P/P	0.0	5.4
P47	European	Italy	M	22.93	29.18	1.38	12.19	0.96	0.56	NO	c.985A>G p.Lys329Glu	c.242-243insT p.Trp82Leufs*23	P/P	0.0	5.1
P48	European	Albania	M	33.72	20.29	1.60	10.19	0.77	0.51	NO	c.985A>G p.Lys329Glu	c.242-243insT p.Trp82Leufs*23	P/P	NA	3.9
P49	European	Albania	M	19.75	24.36	3.15	26.62	1.89	0.25	NO	c.985A>G p.Lys329Glu	c.242-243insT p.Trp82Leufs*23	P/P	NA	4.3
P50	European	Italy	F	12.37	19.41	0.36	0.58	0.44	0.13	NO	c.985A>G p.Lys329Glu	c.271A>G p.Ile91Val	P/VUS	NA	NA
P51	European	Italy	M	47.73	19.64	0.59	3.03	0.31	0.35	NO	c.985A>G p.Lys329Glu	c.200A>G p.Tyr67Cys	P/LP	4.0	NA
P52	European	Italy	F	24.9	17.06	0.47	2.61	0.23	0.18	NO	c.985A>G p.Lys329Glu	c.1167_1168delinsG p.Leu390*	P/LP	2.0	NA
P53	European	Georgia	M	31.34	34.72	0.62	2.33	0.53	0.27	NO	c.985A>G p.Lys329Glu	c.617G>A p.Arg206His	P/LP	21.9	4.1
P54	European	Italy	M	20.76	17.86	1.78	20.22	1.37	0.46	NO	c.985A>G p.Lys329Glu	c.817_829del p.Ala273fs	P/P	NA	3.6
P55	European	Italy	M	18.49	21.41	1.52	9.05	1.24	0.86	Hypoglycemia	c.985A>G p.Lys329Glu	c.388-14 A>G	P/VUS ***	NA	3.9
P56	European	Italy	M	23.86	26.53	1.54	7.70	0.79	0.40	Hypoglycemia	c.985A>G p.Lys329Glu	c.356T>A p.Val119Asp	P/VUS	NA	4.2
P57	European	Italy	F	15.37	16.49	0.39	1.37	1.29	0.18	NO	c.985A>G p.Lys329Glu	c.683C>A p.Thr228Asn	P/VUS	NA	4.2
P58	European	Italy	F	20.86	32.73	1.14	4.24	1.20	0.31	NO	c.985A>G p.Lys329Glu	c.241G>T p.Ala81Ser	P/LP	15.0	NA
P59	European	Italy	M	26.9	29.35	2.12	9.72	1.22	0.45	NO	c.985A>G p.Lys329Glu	c.250C>T p.Leu84Phe	P/P	NA	4.4
P60	African	Morocco	M	22	33.74	0.33	0.79	0.54	0.14	NO	c.985A>G p.Lys329Glu	c.91C>T p.Arg31Cys	P/LP	NA	3.8
P61	European	Italy	M	20.61	26.58	3.35	52.03	3.41	0.78	NO	c.985A>G p.Lys329Glu	c.794_803delinsTTTAA p.Gly265ValfsTer2	P/P	0.03	3.8
P62	European	Italy	F	23.93	21.98	1.24	9.34	0.61	0.39	NO	c.985A>G p.Lys329Glu	c.972delC p.Phe309LeufsTer6	P/P	0.0	5.4
P63	European	Italy	F	13.47	19.26	0.99	6.23	0.69	0.29	NO	c.985A>G p.Lys329Glu	c.1096A>G p.Asn366Asp	P/VUS	0.0	4.2
P64	European	Italy	F	22.87	37.30	0.28	1.17	0.60	0.17	NO	c.985A>G p.Lys329Glu	c.391A>T p.Met131Leu	P/LP-VUS	3.90	4.7
P65	European	Italy	M	25.36	35.91	0.99	4.60	1.72	0.55	NO	c.985A>G p.Lys329Glu	c.698T>C p.Ile233Thr	P/LP	34.10	2.8

^#^ Screening values are not available; the reported values correspond to those measured at the recall visit. Novel variants are indicated by underlining, and their ACMG/AMP classification is provided in the Table. *** Potential Splicing Impact. Not available: enzyme activity measurement not performed. Reference ranges of blood acylcarnitines are expressed in µmol/L. Free carnitine (n.v. 6.6 ± 1.3–54.2 ± 10.2); C2 (n.v. 6.4 ± 2.6–54.3 ± 13.0); C6 (n.v. ≤ 0.2 ± 0.1); C8 (n.v. ≤ 0.3 ± 0.1); C10 (n.v. ≤ 0.3 ± 0.1); C10:1 (n.v. ≤ 0.2 ± 0.1).

**Table 5:** Biochemical newborn screening data in infants compound heterozygous for ACADM variants, other than c.985 A>G (NM_000016.6/NP_000007.1).

Patient	Origin	Country	Sex	Free Carnitine (µmol/L)	C2 (µmol/L)	C6 (µmol/L)	C8 (µmol/L)	C10 (µmol/L)	C10:1 (µmol/L)	Symptoms Within 48 h of Life	Allele 1	Allele 2	ACMG	Enzyme Activity %	Blood Glucose at Recall (n.v. 2.8–6.1 mmol/L)
P66	European	Italy	F	39.1	20.14	0.13	0.27	0.11	0.13	NO	c.799G>A; p.Gly267Arg	c.199T>C; p.Tyr67His	P/P	27.0	4.6
P67	European	Italy	F	11.26	17.51	1.08	8.56	1.02	0.29	NO	c.799G>A; p.Gly267Arg	c.708+2T>G	P/LP	0.0	4.5
P68	Asian	India	M	21.87	30.90	1.06	4.90	1.20	0.47	NO	c.799G>A; p.Gly267Arg	c.946-6T>G	P/VUS	NA	3.6
P69	European	Italy	F	17.37	21.50	0.30	0.50	0.27	0.07	NO	c.242-243insT; p.Trp82Leufs*23	c.50G>A; p.Arg17His	P/VUS	17.0	5.0
P70	European	Albania/ Italy	M	13.68	26.08	0.34	0.73	0.73	0.14	NO	c.242-243insT; p.Trp82Leufs*23	c.1091T>C; p.Phe364Ser	P/VUS	29.0	2.3
P71	European	Italy	M	22.17	44.43	0.56	1.27	0.91	0.17	NO	c.242-243insT; p.Trp82Leufs*23	c.599+3A>G	P/VUS	34.0	3.9
P72	European	Italy	F	11.26	28.53	1.21	4.19	1.55	0.35	NO	c.242-243insT; p.Trp82Leufs*23	c.1207A>G; p.Thr403Ala	P/VUS	NA	3.8
P73	European	Italy	F	13.70	20.20	1.35	13.09	1.11	0.41	NO	c.242-243insT; p.Trp82Leufs*23	c.1102_1105delTTAG; p.Ala369Leufs*18	P/P	2.0	4.2
P74	European	Italy	M	17.73	10.83	0.46	1.33	0.57	0.28	NO	c.797A>G; p.Asp266Gly	c.333delA; p.Glu111AspfsTer39	LP/LP	21.0	3.9
P75	European	Italy	F	20.16	25.47	0.47	0.46	0.64	0.20	NO	c.797A>G; p.Asp266Gly	c.708+2T>G	LP/LP	27.0	3.9
P76	European	Italy	F	17.90	15.40	0.74	3.00	1.05	0.73	NO	c.797A>G; p.Asp266Gly	c.387+1del	LP/P	24.0	3.4
P77	European	Italy	M	17.50	26.70	0.57	1.39	0.64	0.33	NO	c.797A>G; p.Asp266Gly	c.387+1del	LP/P	24.0	5.5
P78	European	Italy	F	15.93	16.80	0.26	0.58	0.42	0.15	NO	c.797A>G; p.Asp266Gly	c.817_829del; p.Ala273LeufsTer7	LP/P	17.2	4.1
P79	European	Italy	F	21.9	19.87	0.27	0.93	0.64	0.17	NO	c.253G>C; p.Gly85Arg	c.734C>T; p.Ser245Leu	P/P	28.0	2.2
P80	European	Italy	F	18.30	23.30	0.16	0.29	0.25	0.11	NO	c.253G>C; p.Gly85Arg	c.599+3A>G	P/VUS	NA	NA
P81	European	Italy	M	29.8	12.99	0.667	7.26	0.515	0.58	NO	c.253G>C; p.Gly85Arg	c.279_280del; p.Asn94LeufsTer10	P/LP	4.0	4.1
P82	European	Italy	M	17.00	8.8	0.32	1.56	0.21	0.17	NO	c.168delC; p.Arg57fs	c.926T>G; p.Phe309Cys	P/LP	NA	2.7
P83	European	Italy	F	40.46	28.30	0.50	0.90	0.30	0.16	NO	c.50G>C; p.Arg17Pro	c.512G>T; p.Gly171Val	VUS/VUS	12.2	3.8
P84	European	Italy	F	39.00	34.40	1.10	9.30	0.70	0.60	NO	c.421C>T; p.Gln141Ter	c.1096A>G; p.Asn366Asp	LP/LP	0.0	3.1
P85	south American	Chile	F	10.07	15.72	0.16	0.44	0.49	0.09	NO	c.250C>T; p.Leu84Phe	c.683C>A; p.Thr228Asn	P/LP	NA	3.6
P86	European	Italy	F	13.10	36.09	0.48	1.72	1.06	0.24	NO	c.1207A>G; p.Thr403Ala	c.449C>A; p.Thr150Asn	VUS/VUS	22.0	5.1
P87	African	Morocco	M	8.31	10.32	2.42	15.60	2.22	0.58	hyporeactivity, hypoglycemia	c.362C>T; p.Thr121Ile	c.317G>A; p.Cys106Tyr	P/LP	NA	5.0
P88	European	Italy	F	18.70	25.75	0.69	2.38	1.14	0.20	NO	c.199T>C p.Tyr67His	c.200A>G p.Tyr67Cys	P/P	NA	4.0

Novel variants are indicated by underlining, and their ACMG/AMP classification is provided in the Table. Not available: enzyme activity measurement not performed. Reference ranges of blood acylcarnitines are expressed in µmol/L. Free carnitine (n.v. 6.6 ± 1.3–54.2 ± 10.2); C2 (n.v. 6.4 ± 2.6–54.3 ± 13.0); C6 (n.v. ≤ 0.2 ± 0.1); C8 (n.v. ≤ 0.3 ± 0.1); C10 (n.v. ≤ 0.3 ± 0.1); C10:1 (n.v. ≤ 0.2 ± 0.1).

## Data Availability

The original contributions presented in this study are included in the article/Supplementary Material. Further inquiries can be directed to the corresponding author(s).

## Supplementary Materials

The following supporting information can be downloaded at https://www.mdpi.com/2409-515X/11/4/86/s1: Figure S1: MCAD residual activity versus case numbers; Figure S2: Distribution of C0 levels on DBS samples from subjects stratified by MCAD residual activity expressed as percentage of wt.

## Appendix A

Lucia Albano ^2^, Luisella Alberti ^3^, Concetta Aloi ^10^, Antonio Angeloni ^4^, Maurizio Aricò ^11^, Federico Baronio ^12^, Ferdinando Barretta ^2^, Marta Camilot ^7,8^, Claudia Carducci ^4^, Michela Cassanello ^13^, Cinzia Castana ^14^, Chiara Cazzorla ^9^, Graziella Cefalo ^15^, Renzo Ciatti ^16^, Daniela Concolino ^17^, Daniela Crisci ^2^, Donatella De Giovanni ^18^, Carlo Dionisi-Vici ^6^, Giulia Frisso ^1,2^, Silvia Funghini ^5^, Daniela Gasperini ^19^, Serena Gasperini ^20^, Stefania Graci ^21^, Anastasia Ibba ^19^, Giancarlo la Marca ^5,22^, Angelico Lampis ^19^, Annalisa Lonetti ^23^, Vincenza Gragnaniello ^9^, Luisa La Spina ^24^, Francesca Romana Lepri ^25^, Giuseppe Lippi ^26^, Simona Lucchi ^3^, Onorina Marasco ^27^, MariaAnna Messina ^24^, Amelia Morrone ^28,29^, Giorgia Olivieri ^6^, Giancarlo Parenti ^30^, Enza Pavanello ^31^, Michela Perrone Donnorso ^32,33^, Andrea Pession ^34^, Elena Porcù ^9^, Francesco Porta ^35^, Elena Procopio ^36^, Francesca Righetti ^23^, Cristiano Rizzo ^6^, Claudia Rossi ^37^, Laura Rubert ^38^, Martino Ruggieri ^24,39^, Silvia Russo ^40^, Leonardo Salviati ^41^, Laura Santoro ^21^, Pina Sauro ^35^, Giovanna Scozzafava ^27^, Simonetta Simonetti ^40^, Marco Spada ^35^, Francesco Tagliaferri ^42^, Elisabetta Tarsi ^16^, Giulia Tozzi ^6^, Albina Tummolo ^18^, Alessandra Vasco ^3^, Monica Vincenzi ^8^

1. Department of Molecular Medicine and Medical Biotechnology, University of Naples, Federico II, 80131 Naples, Italy
2. Ceinge Advanced Biotechnology Franco Salvatore Scarl, 80131 Naples, Italy
3. Center of Functional Genomics and Rare Diseases, ‘V. Buzzi’ Children’s Hospital, 20154 Milan, Italy
4. Department of Experimental Medicine, Newborn Screening Center-Clinical Pathology Unit, University Hospital Policlinico Umberto I, Sapienza University, 00161 Rome, Italy
5. Newborn Screening, Clinical Biochemistry and Clinical Pharmacy Laboratory, Meyer Children’s Hospital IRCCS, 50139 Florence, Italy
6. Division of Metabolic Diseases and Hepatology, Bambino Gesù Children’s Hospital, IRCCS, 00165 Rome, Italy
7. Department of Surgery, Dentistry, Paediatrics and Gynaecology, University of Verona, 37129 Verona, Italy
8. Laboratory Medicine Service, Regional Center for Newborn Screening, Diagnosis and Treatment of Inherited Metabolic Diseases and Congenital Endocrine Diseases, Azienda Ospedaliera Universitaria Integrata of Verona, 37126 Verona, Italy
9. Division of Inherited Metabolic Diseases, Department of Women’s and Children’s Health, University Hospital of Padua, 35128 Padua, Italy
10. LABSIEM, Laboratory for the Study of Inborn Errors of Metabolism, Pediatric Clinic, IRCCS Istituto Giannina Gaslini, 16147 Genoa, Italy
11. U.O.C. Pediatrics, S. Spirito Hospital, ASL Pescara, 65124 Pescara, Italy
12. Pediatric Unit, IRCCS Azienda Ospedaliero-Universitaria di Bologna, 40138 Bologna, Italy
13. Biochemistry, Pharmacology and Newborn Screening Unit, Central Laboratory of Analysis, IRCCS Istituto Giannina Gaslini, 16147 Genoa, Italy
14. U.O.C. General Pediatrics, U.O.S. Diabetology and MME CCR-SNE, G. di Cristina Children’s Hospital, 90134 Palermo, Italy
15. Pediatric Unit, Department of the Woman and Child, ASST Santi Paolo e Carlo, San Paolo Hospital—, University of Milan, 20142 Milan, Italy
16. Newborn Screening Center, OUC Pediatric Neuropsychiatry, Azienda Sanitaria Territoriale, PESARO-URBINO, Stabilimento Santacroce, 61032 Fano, Italy
17. Department of Health Science, university of Magna Grecia University, 88100 Catanzaro, Italy
18. Department of Metabolic Diseases, Clinical Genetics Giovanni XXIII Children Hospital, Azienda Ospedaliero-Universitaria Consorziale, 70126 Bari, Italy
19. SSD Pediatric Endocrinology and Neonatal Screening Center, “A. Cao” Microcitemic Children’s Hospital, ASL CAGLIARI, 09121 Cagliari, Italy
20. Pediatrics, Fondazione IRCCS San Gerardo dei Tintori, 20900 Monza, Italy
21. Department of Advanced Diagnostics,—U.O.S. Pediatric Clinical Pathology CSNE Lab, G. di Cristina Pediatric Hospital, 90134 Palermo, Italy
22. Department of Experimental and Clinical Biomedical Sciences “Mario Serio”, University of Florence, 50134 Florence, Italy
23. Regional Laboratory for Neonatal Screening and Endocrine-Metabolic Diseases, IRCCS University Sant’Orsola Hospital, 40138 Bologna, Italy
24. Expanded Newborn Screening and Metabolic Diseases Unit, AOU Policlinico “G. Rodolico-San Marco”, PO “G. Rodolico, 95123 Catania, Italy
25. Laboratory of Medical Genetics, Translational Cytogenomics Research Unity, Bambino Gesù Children’s Hospital, IRCCS, 00165 Rome, Italy
26. Section of Clinical Biochemistry, Department of Engineering for Innovative Medicine (DIMI), University of Verona, 37134 Verona, Italy
27. AOU Mater Domini, 88100 Catanzaro, Italy
28. Laboratory of Molecular Genetics of Neurometabolic Diseases, Neuroscience and HumanGenetics Department, Meyer Children’s Hospital IRCCS, 50139 Florence, Italy
29. Department of Neuroscience, Psychology, Drug Research and Child Health (NEUROFARBA), University of Florence, 50139 Florence, Italy
30. Department of Translational Medicine, University of Naples “Federico II”, 80131 Naples, Italy
31. SS Prenatal and Neonatal Screening, SC Clinical Biochemistry, AOU Città della Salute e della Scienza di Torino, 10126 Turin, Italy
32. LABSIEM (Laboratory for the Study of Inborn Errors of Metabolism), Pediatric Clinic, DINOGMI (Department of Neuroscience, Rehabilitation, Ophthalmology, Genetics, Maternal and Child Health), University of Genova, 16132 Genoa, Italy
33. Department of Pediatrics, IRCCS Giannina Gaslini, 16147 Genoa, Italy
34. Department of Medical and Surgical Sciences, Alma Mater Studiorum, University of Bologna, 40138 Bologna, Italy
35. Department of Pediatrics, University of Torino, 10126 Turin, Italy
36. Metabolic and Muscular Unit, Meyer Children’s Hospital IRCCS, 50139 Florence, Italy
37. Department of Science, Center for Advanced Studies and Technology (CAST), “G. d’Annunzio” University of Chieti-Pescara, 66100 Chieti, Italy
38. Department of Pediatrics, Regional Center for Newborn Screening, Diagnosis and Treatment of Inherited Metabolic Diseases and Congenital Endocrine Diseases, Azienda Ospedaliera Universitaria Integrata of Verona, 37126 Verona, Italy
39. Unit of Pediatric Clinic and Department of Clinical and Experimental Medicine, AOU Policlinico “G. Rodolico-San Marco”, PO “G. Rodolico, 95123 Catania, Italy
40. Department of Clinical Pathology and Neonatal Screening Giovanni XXIII Children Hospital Azienda Ospedaliero-Universitaria Consorziale, 70126 Bari, Italy
41. Clinical Genetics Unit, Department of Women’s and Children’s Health, University Hospital of Padua, 35128 Padua, Italy
42. Fondazione IRCCS Ca’ Granda Ospedale Maggiore Policlinico, Clinical Metabolic Reference Center, 20122 Milan, Italy
