# Supplementary material for: Medium-Chain Acyl-CoA Dehydrogenase Deficiency (MCADD) Newborn Screening in Italy: Five Years’ Experience from a Nationwide Program
Source: Int J Neonatal Screen. 2025 Sep 26;11(4):86. doi: 10.3390/ijns11040086 (PMC12550940; doi:10.3390/ijns11040086)
Supplement: Supplementary file 1 [file IJNS-11-00086-s001.zip › IJNS-3754467-supplementary.pdf]

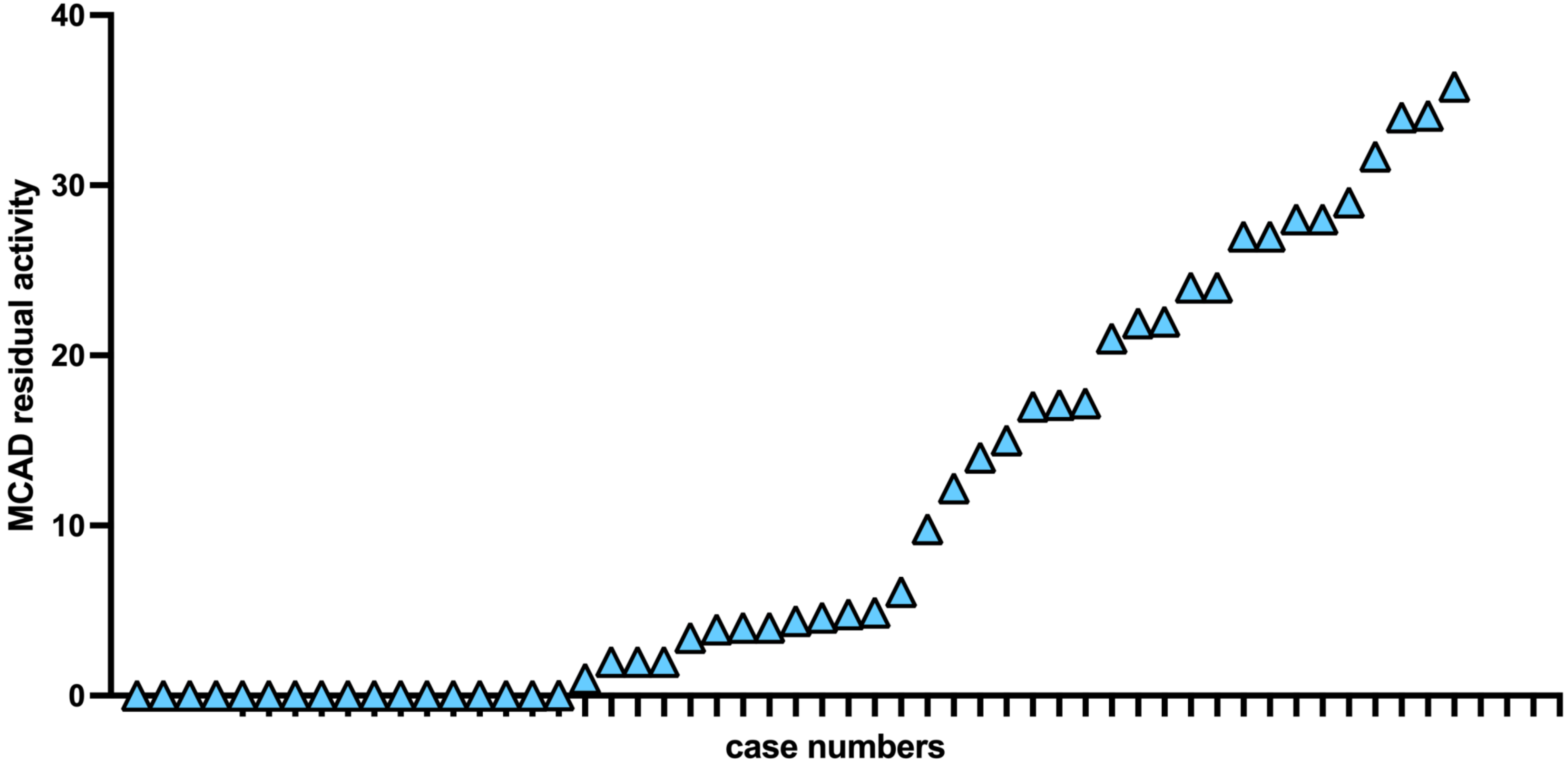

Figure S1. MCAD residual activity versus case numbers.

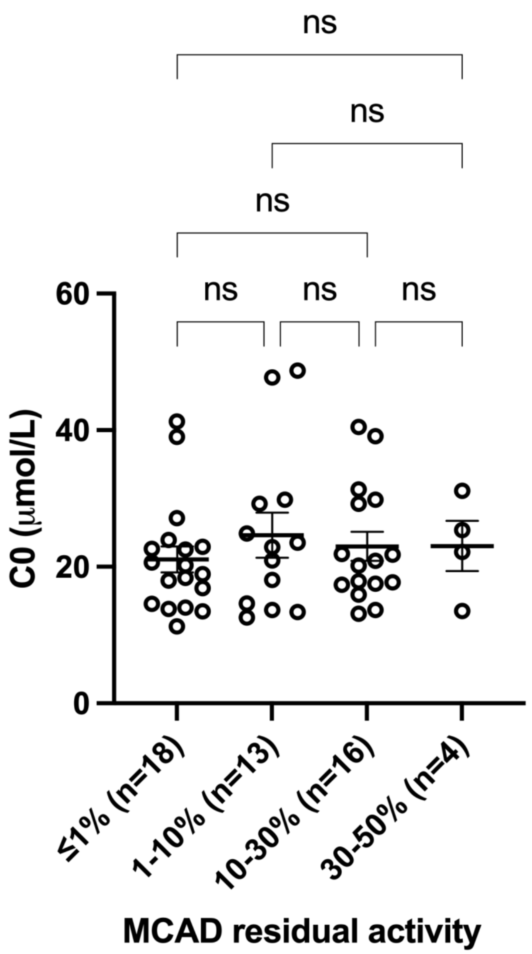

Figure S2. Distribution of C0 levels on DBS samples from subjects stratified by MCAD residual activity expressed as percentage of wt.
